# Functional brain-specific microvessels from iPSC-derived human brain microvascular endothelial cells: the role of matrix composition on monolayer formation

**DOI:** 10.1186/s12987-018-0092-7

**Published:** 2018-02-20

**Authors:** Moriah E. Katt, Raleigh M. Linville, Lakyn N. Mayo, Zinnia S. Xu, Peter C. Searson

**Affiliations:** 1 Institute for Nanobiotechnology, Johns Hopkins University, Baltimore, MD USA; 2 Department of Materials Science and Engineering, Johns Hopkins University, Baltimore, MD USA; 3 Department of Biomedical Engineering, Johns Hopkins University, Baltimore, MD USA

**Keywords:** Brain microvascular endothelial cells, Stem cells, Transendothelial electrical resistance, Microvessels, Tissue-engineering

## Abstract

**Background:**

Transwell-based models of the blood–brain barrier (BBB) incorporating monolayers of human brain microvascular endothelial cells (dhBMECs) derived from induced pluripotent stem cells show many of the key features of the BBB, including expression of transporters and efflux pumps, expression of tight junction proteins, and physiological values of transendothelial electrical resistance. The fabrication of 3D BBB models using dhBMECs has so far been unsuccessful due to the poor adhesion and survival of these cells on matrix materials commonly used in tissue engineering.

**Methods:**

To address this issue, we systematically screened a wide range of matrix materials (collagen I, hyaluronic acid, and fibrin), compositions (laminin/entactin), protein coatings (fibronectin, laminin, collagen IV, perlecan, and agrin), and soluble factors (ROCK inhibitor and cyclic adenosine monophosphate) in 2D culture to assess cell adhesion, spreading, and barrier function.

**Results:**

Cell coverage increased with stiffness of collagen I gels coated with collagen IV and fibronectin. On 7 mg mL^−1^ collagen I gels coated with basement membrane proteins (fibronectin, collagen IV, and laminin), cell coverage was high but did not reliably reach confluence. The transendothelial electrical resistance (TEER) on collagen I gels coated with basement membrane proteins was lower than on coated transwell membranes. Agrin, a heparin sulfate proteoglycan found in basement membranes of the brain, promoted monolayer formation but resulted in a significant decrease in transendothelial electrical resistance (TEER). However, the addition of ROCK inhibitor, cAMP, or cross-linking the gels to increase stiffness, resulted in a significant improvement of TEER values and enabled the formation of confluent monolayers.

**Conclusions:**

Having identified matrix compositions that promote monolayer formation and barrier function, we successfully fabricated dhBMEC microvessels in cross-linked collagen I gels coated with fibronectin and collagen IV, and treated with ROCK inhibitor and cAMP. We measured apparent permeability values for Lucifer yellow, comparable to values obtained in the transwell assay. During these experiments we observed no focal leaks, suggesting the formation of tight junctions that effectively block paracellular transport.

**Electronic supplementary material:**

The online version of this article (10.1186/s12987-018-0092-7) contains supplementary material, which is available to authorized users.

## Background

Brain microvascular endothelial cells (BMECs) are highly specialized with tight junctions that effectively eliminate paracellular transport, transporters to deliver essential nutrients to the brain, and efflux pumps to transport unwanted substrates back into circulation [1, 2]. The lack of physiologically relevant BMEC lines has been a major roadblock to blood–brain barrier (BBB) research [3], however, stem cell technology has provided a potential solution to this problem [4].

Human induced pluripotent stem cells (hiPSC) have been used extensively to study cells with neuronal lineages in both health and disease [5–7]. More recently iPSCs have been differentiated into brain microvascular endothelial cells (dhBMECs) from a number of iPSC lines including: BC1 [8], IMR90-4 [4, 9–11], ARiPS [11], DF6-9-9T [12], DF19-9-11T [12], H9 embryonic stem cells, as well as patient lines from Huntington’s disease [13]. All of these iPSC lines produce dhBMECs with characteristics of the BBB, including high transendothelial electrical resistance (TEER), greater than 1000 Ω cm^2^ for cells from healthy individuals, claudin-5- and occludin-positive tight junctions, polarized P-gp efflux, ≥ 90% endothelial purity, and many other important characteristics of the BBB [4, 8–15]. Therefore, differentiated iPSCs play an important role in BBB research since they provide a renewable and reproducible source of human BMECs.

Accumulating evidence suggests that in addition to their barrier function, human brain microvascular endothelial cells exhibit other unique characteristics that contribute to their phenotype. For example, in cell culture hBMECs and dhBMECs do not undergo a transition from cobblestone to spindle morphology under shear flow [16, 17]. Similarly hBMECs and dhBMECs do not elongate and align in response to the high curvature of capillary dimensions [8, 18].

While 2D transwell models provide an important tool in BBB research [1, 19], recent attention has focused on the fabrication of 3D models that recapitulate the cylindrical microvessel geometry and shear flow. Advances in tissue engineering have enabled fabrication of functional endothelial microvessels in a gel matrix [20–25], however, attempts to recapitulate the key characteristics of the BBB using primary or immortalized BMECs have had limited success [26–28]. Stem-cell derived hBMECs provide a potential solution to this problem, although incorporation of dhBMECs into tissue-engineered BBB models has been challenging since dhBMECs are less adherent and proliferative than other endothelial cells.

The objective of this work was to identify matrix compositions for the formation of confluent dhBMEC monolayers and maintenance of barrier function. We first screened adhesion and spreading of dhBMECs on 2D collagen I gels as a function of gel stiffness, composition (collagen I and collagen I + laminin/entactin), surface modification (fibronectin, laminin, collagen IV, perlecan, and agrin), and the addition of soluble factors [ROCK inhibitor Y-27632 and cyclic adenosine monophosphate (cAMP)]. We then assessed barrier function of dhBMEC monolayers on porous supports with different coatings or bulk hydrogels. Finally, having identified conditions that promote formation of confluent monolayers with physiological barrier properties in 2D, we then demonstrated the formation of perfusable brain-specific microvessels with confluent monolayers of dhBMECs in a genipin—crosslinked collagen I matrix coated with fibronectin and collagen IV with the addition of the ROCK inhibitor Y-27632 and cAMP in the perfusion media during seeding.

## Methods

### Cell culture and differentiation

BC1 GFP iPSCs were cultured and differentiated as previously reported [8]. Briefly, iPSCs were cultured on matrigel-coated tissue culture flasks (Corning, Corning, New York, USA) in TeSR-E8™ medium (StemCell Technologies, Vancouver, BC, Canada) to 50% confluence. Then the media was switched to unconditioned media without basic fibroblast growth factor [DMEM/F12 supplemented with 80% KOSR, 1% non-essential amino acids, 0.5% glutaMAX, and 0.836 µM beta-mercaptoethanol (Life Technologies, Carlsbad, California, USA)] for 7 days, then transferred to endothelial cell medium [endothelial cell serum free medium, 1% human platelet poor-derived serum, 20 ng mL^−1^ bFGF (R&D Systems, Minneapolis, Minnesota, USA), 10 µM retinoic acid (Sigma, St. Louis, Missouri, USA)]. Cells were then sub-cultured on collagen IV and fibronectin coated plates for 1 h before being subsequently passaged onto the experimental substrate at a density of 1 × 10^6^, or 5 × 10^6^ cells mL^−1^ on transwells. In previous work we have shown that confluent monolayers are formed on collagen IV and fibronectin-coated glass and transwell supports at this density [8]. The medium was changed 24 h following subculture and every 48 h after that. Where indicated cells were treated with 10 µM ROCK inhibitor (Y-27632) (ATCC, Manassas, Virginia, USA) for the first 24 h and 100 µM dibutyryl cyclic AMP (Sigma) throughout culture following subculture.

### Matrix formation

Unless otherwise stated, gels were fabricated from collagen I (Corning) ranging from 4 to 7 mg mL^−1^ neutralized with sodium hydroxide. Laminin/entactin (Corning) was added to collagen I gels at densities of 0.25–1 mg mL^−1^. Collagen I gels were formed at 37 °C for 30 min and then coated with 50 µg mL^−1^ of proteins and heparan sulfate proteoglycans at 37 °C overnight. The thickness of the gels was 300–500 µm. Where indicated, the gels were cross-linked with 20 mM genipin (Sigma) for 2 h and washed with PBS for 24–48 h at room temperature before coating. Genipin is a low molecular weight cross-linker that has been used previously to enhance vascular stability and increases the stiffness of collagen fivefold [29]. Collagen I gels were coated with collagen IV (Sigma), fibronectin (Life Technologies), laminin (Sigma), agrin (R&D Systems), and/or perlecan (R&D Systems).

### Immunofluorescence

To image monolayers, the gels were fixed and stained, then inverted on a glass slide. Four days after seeding, gels were washed with PBS then fixed in 3.7% paraformaldehyde for 10 min. Cells were then permeablized for 1 h in 0.1% Triton-X100 and blocked for 1 h in 10% donkey serum in PBS. Primary antibodies were incubated overnight at 4 °C in 10% donkey serum followed by 1 h incubation with secondary antibodies in 10% donkey serum. Cells were stained for ZO-1 (Life Technologies), claudin-5 (Life Technologies), and DAPI as described previously [8]. Imaging was performed on a Nikon TiE confocal microscope using 40× objective and NIS Advanced Research software (Nikon, Minato, Tokyo, Japan). The images are maximum intensity projections of confocal z-stacks with a z spacing of 0.4 µm.

### Transendothelial electrical resistance

Transendothelial electrical resistance (TEER) was measured daily for 1 week after seeding on polyester 12-well transwell membranes with a 0.4 µm pore size (Corning), as previously described [8]. TEER measurements were recorded using a STX2 probe with EVOM2 (World Precision Instruments, Sarasota, Florida, USA). Peak TEER values on coated transwells were typically recorded between days 2 and 4 and were used for comparison to experiments where monolayers were formed on bulk gels (300–500 µm thick) in the transwell inserts, as described above. Note that TEER values on 12-well transwell inserts are typically 2.5-fold higher than the values of around 2000 Ω cm^2^ reported on 24-well inserts [8, 10]. All TEER experiments were performed in triplicate (3 wells) for each of three distinct differentiations.

### Cell coverage and junctional analysis

The endothelial cell coverage on different substrates was measured from phase contrast images of cells on coated gels daily for 3 days. Images were taken following a wash and media exchange step to ensure only adherent cells were considered in analysis. Images were taken in three randomly selected areas of the plate for three separate differentiations, resulting in at least nine images per condition. Images were then analyzed using Image J to determine the percent coverage. Cell area was calculated from maximum intensity ZO-1 projection images manually using ImageJ on at least 75 cells per condition. Measurements of cell–cell junction width were obtained from claudin-5 confocal z-stack images with a slice height of 0.4 µm from at least 150 junctions. Slices where the claudin-5 junctions were clear and in focus, which ranged from 3 to 10 slices, were stacked and the width of the junction in the collective images were measured in NIS Advanced Research software (Additional file 1: Figure S1).

### Statistical analysis

Statistical differences in coverage and TEER were determined using one-way ANOVA, with Tukey’s multiple comparisons test. P values of less than 0.05 were considered statistically significant (*), with P ≤ 0.01 represented with **, and P ≤ 0.001 represented with ***.

### Microvessel fabrication and characterization

Microvessels were fabricated as previously reported [22]. Briefly, neutralized 7 mg mL^−1^ collagen I was injected into a polydimethylsiloxane and glass enclosure around a 150 µm diameter Nitinol template rod and allowed to gel at 37 °C. 2% agarose was injected at the ends of the collagen I to prevent delamination. After template rod removal, channels were cross-linked with 20 mM genipin for 2 h and washed with PBS for 24–48 h. The platform was then treated overnight with endothelial cell medium containing 50 µg mL^−1^ fibronectin and 100 µg mL^−1^ collagen IV at 37 °C. Endothelial cell media was supplemented with 10 µM retinoic acid, 400 µM dibutyryl cyclic AMP, 20 µM phosphodiesterase inhibitor Ro-20-1724 (Sigma), and 3% 70 kDa dextran (Sigma) to promote vascular stability [29–31]. dhBMECs were introduced into the channel at a density of 6 × 10^7^ cells mL^−1^ under low flow and allowed to settle and adhere for 1 h. Low shear flow (~ 0.1 dyn cm^−1^) was applied for the first 24 h; during this time the media was additionally supplemented with 10 µM ROCK inhibitor. Flow was increased after 24 h to 1.0 dyn cm^−1^.

Three days after seeding, the permeability of the microvessels to Lucifer yellow was measured using methods reported previously [22, 32]. 100 µM Lucifer yellow (Sigma) was perfused through microvessels for 45 min, and fluorescence images recorded every 5 min. Images were analyzed using ImageJ, and apparent permeability was calculated from Papp = (d/4)(1/∆I)(dI/dt) where d is the vessel diameter, ΔI is the initial increase in fluorescence intensity due to perfusion in the lumen, and (dI/dt) is the rate of increase in fluorescence intensity. Imaging was performed on a Nikon TiE microscope using 10× objective and NIS Advanced Research software. Permeability was measured in microvessels seeded from three distinct differentiations.

## Results

### Extracellular matrix (ECM)

To assess cell adhesion and spreading, dhBMECs were seeded onto gels with different composition, stiffness, and coatings. To compare adhesion across different conditions we measured the coverage of the dhBMECs over the first 72 h of culture. In general, cells either adhered to the matrix and formed monolayers, or showed poor adhesion and formed small mounds that eventually detached from the matrix (Additional file 1: Figure S2).

### ECM composition and concentration

**Fig. 1:**
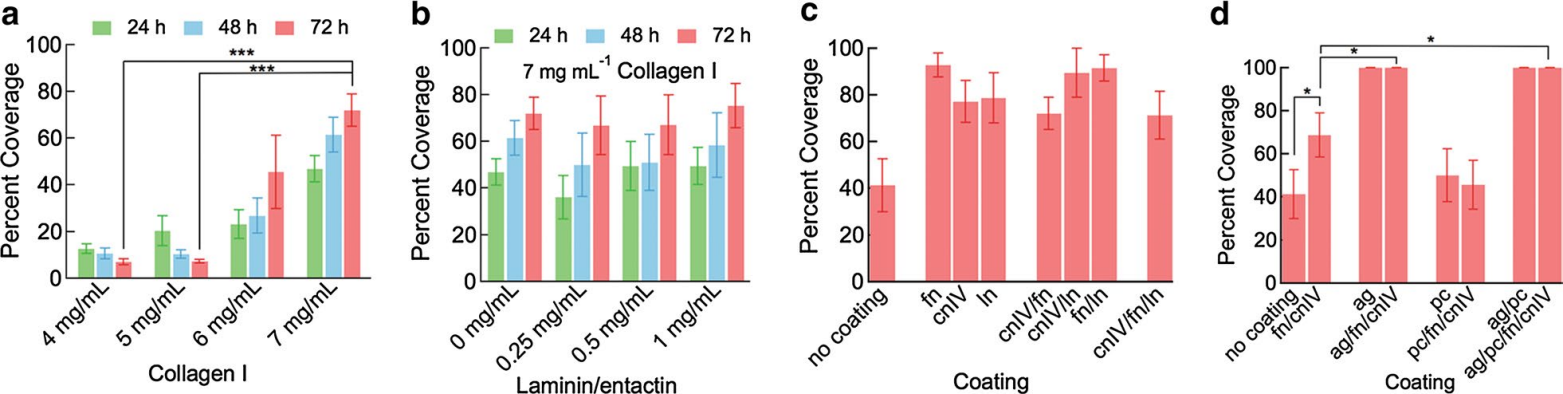
The influence of ECM on monolayer formation. **a** Cell coverage on varying concentrations of collagen I gels coated with fibronectin and collagen IV at 24, 48, and 72 h following seeding. **b** Cell coverage on 7 mg mL^−1^ collagen I gels supplemented with laminin/entactin and coated with fibronectin and collagen IV. **c** Coverage on 7 mg mL^−1^ collagen I gel coated with the proteins indicated fn—fibronectin, cnIV—collagen IV, ln—laminin. All coatings result in significantly higher coverage compared to uncoated collagen I gels (P < 0.05), with no difference between different coatings (P > 0.05). **d** Coverage on 7 mg mL^−1^ collagen I gel coated with selected proteins (fn—fibronectin, cnIV—collagen IV) and selected heparan sulfate proteoglycans (ag—agrin, pc—perlecan). Bars represent mean ± standard error, * represents p < 0.05, *** represents p < 0.001

To assess the role of ECM stiffness on monolayer formation, dhBMECs were seeded onto collagen I gels coated with fibronectin and collagen IV. The fractional coverage of dhBMECs was strongly dependent on ECM stiffness, with coverages ranging from 7 ± 1% on 4 mg mL^−1^ gels to 72 ± 7% on 7 mg mL^−1^ gels 72 h after seeding (Fig. 1a). For the lowest collagen concentrations (4 and 5 mg mL^−1^), the cell coverage decreased with time as mounds of cells coalesced on the surface. In contrast to the dhBMECs, other widely used human- and animal-derived ECs form confluent monolayers when seeded onto collagen I gels in this concentration range [22–24, 33, 34]. For the highest collagen concentrations (6 and 7 mg mL^−1^), the cell coverage increased with time due to spreading and proliferation.

We assessed monolayer formation on 7 mg mL^−1^ collagen I gels with the addition of 0.25, 0.5, 0.75, and 1 mg mL^−1^ laminin/entactin and coated with collagen IV and fibronectin (Fig. 1b). The laminin concentrations were based on previous studies of neuronal outgrowth and cerebral organoid development [35, 36]. The addition of laminin/entactin did not change the cell coverage, even at the highest concentration (Fig. 1b). In each case the coverage increased with time, up to around 70% after 72 h, similar to the control with no laminin/entactin.

### Basement membrane components: fibronectin, laminin, collagen IV, perlecan, and agrin

To assess the role of basement membrane proteins on adhesion and spreading in more detail, 7 mg mL^−1^ collagen gels were coated with combinations of basement membrane proteins. Monolayers formed on collagen I gels coated with combinations of basement membrane proteins were not fully confluent, leaving large openings in the monolayer that did not close following 3 days of culture. However, in all cases, coating with basement membrane proteins resulted in higher cell coverage compared to the control with no coating (Fig. 1c). There was no statistical difference in the area covered by the cells between coating with one, two, or three components.

Coating collagen I gels with heparan sulfate proteoglycans resulted in a highly variable propensity for monolayer formation (Fig. 1d). On gels coated with perlecan or a combination of perlecan, fibronectin, and collagen IV, dhBMECs showed poor adhesion, similar to the uncoated control. In contrast, on gels with agrin or a combination of agrin, fibronectin, and collagen IV, complete monolayers were formed. Monolayer formation was also observed on gels coated with agrin and perlecan or a combination of agrin, perlecan, fibronectin, and collagen IV. These results suggest that agrin is important in promoting adhesion of dhBMECs.

### Barrier function

**Fig. 2:**
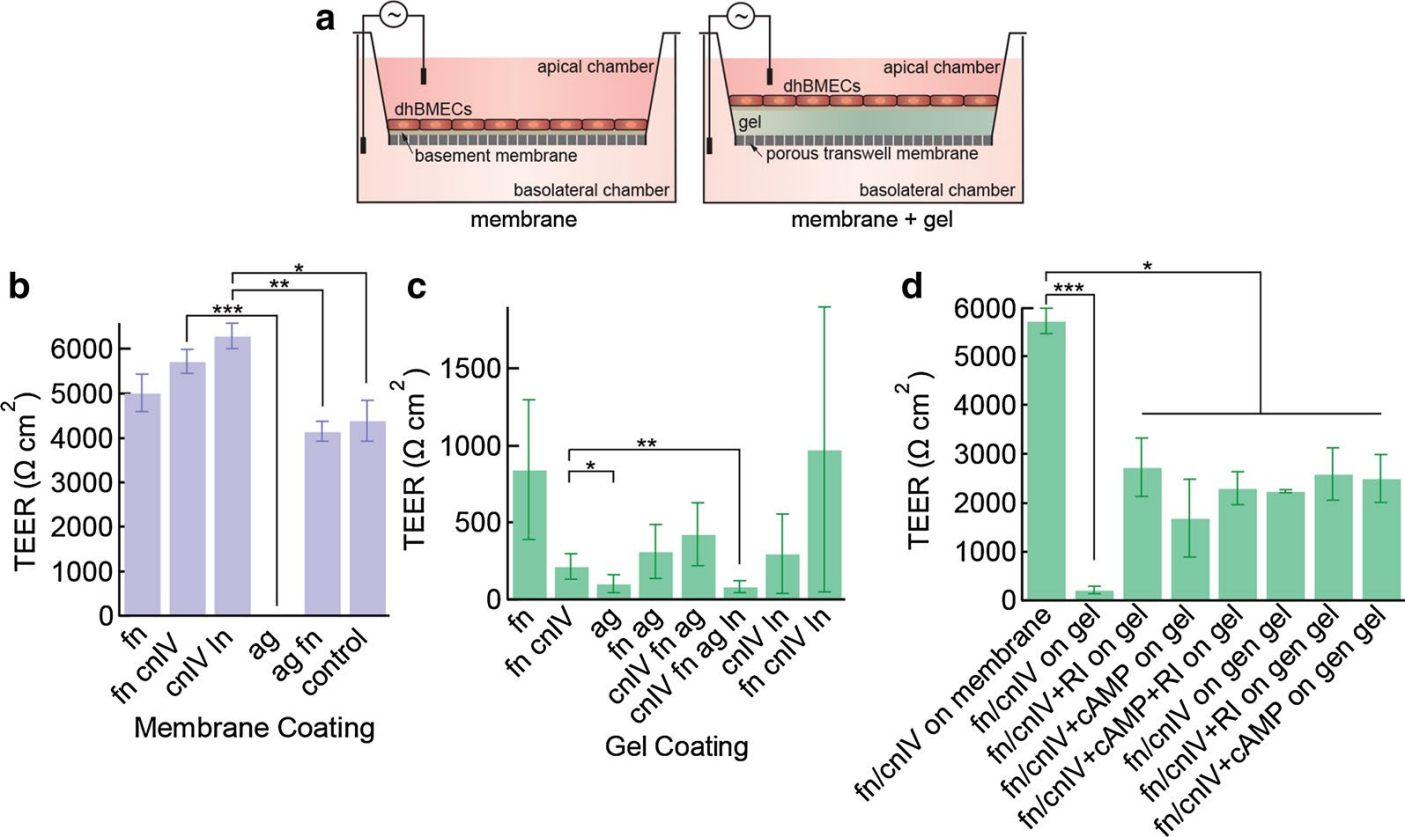
Barrier function of dhBMEC monolayers. **a** Schematic illustration of transwell assay for TEER measurements for dhBMEC monolayers on coated membranes or collagen I gels and membrane coatings. **b** Peak TEER on membranes coated with basement membrane proteins and heparan sulfate proteoglycans that resulted in monolayer formation coverage where the control is cells directly subcultured onto fn + cnIV coated membranes, compared to cells subcultured on plastic tissue culture plates coated with collagen IV and fibronectin before plating onto coated transwells. **c** Peak TEER taken on membranes coated with collagen gels and basement membrane coating mixtures. **d** Peak TEER on transwells with collagen gels coated with fibronectin and collagen IV, and different combinations of ROCK inhibitor Y27632, cAMP, and genipin treatment. Bars represent mean ± standard error, * represents p < 0.05, ** represents p < 0.01, *** represents p < 0.001

Cell adhesion and spreading is important for monolayer formation, but does not necessarily confirm barrier function. The formation of tight junctions minimizes paracellular transport, a key feature of the BBB. To assess barrier function we measured the transendothelial electrical resistance (TEER) of dhBMEC monolayers on transwell membranes and on transwell membranes modified with bulk collagen I gels (Fig. 2a).

### Barrier function of monolayers on transwell membranes coated with basement membrane components

TEER values for dhBMEC monolayers on transwell membranes coated with fibronectin and collagen IV were around 4400 Ω cm^2^ 48 h after seeding, and were used as the control. To avoid the influence of the different coatings on dhBMEC purification/selection, cells were sub-cultured in tissue culture flasks coated with collagen IV and fibronectin for 1 h before seeding on the coated transwell membranes. After the subculture step, TEER values of dhBMEC monolayers on membranes coated with fibronectin and collagen IV were around 5700 cm^2^. hBMEC monolayers on membranes coated with fibronectin, fibronectin and collagen IV, or collagen IV and laminin showed similarly high TEER values (Fig. 2b). In contrast, the TEER of dhBMECs on agrin-coated membranes was 5 Ω cm^2^, significantly lower than the control. These results show that the formation of a confluent monolayer is not sufficient to confirm barrier formation since agrin promotes monolayer formation (Fig. 1d), but these monolayers failed to develop barrier properties, as indicated by the low TEER values. However, monolayers on membranes coated with agrin and fibronectin show similar TEER to fibronectin-coated membranes and the control.

### Barrier function of monolayers on collagen I gels

To assess barrier function of monolayers under conditions relevant for 3D, we measured TEER values of monolayers on collagen I gels on the transwell membranes (Fig. 2c, Additional file 1: Figure S3). Cell monolayers on fibronectin-coated gels showed TEER values of 840 Ω cm^2^, significantly lower than on the transwell membranes. Similar TEER values were obtained for gels coated with fibronectin, collagen IV, and laminin. Monolayers on gels coated with agrin, exhibited TEER values about 100 Ω cm^2^. Monolayers on gels coated with agrin and other basement membrane components exhibited TEER values from 80 to 400 Ω cm^2^.

### Effects of soluble factors on the barrier properties of the monolayers

Based on the results from screening matrix materials and coatings, collagen I gels (7 mg mL^−1^) coated with fibronectin and collagen IV show a good combination of monolayer formation and barrier function, although the TEER values were about fivefold lower than on fibronectin-coated transwell membranes. Soluble factors can also modulate adhesion, spreading, and barrier function, and hence we assessed two molecules: the ROCK inhibitor Y-27632 (RI) and cyclic adenosine monophosphate (cAMP). RI did not significantly increase adhesion to the collagen I gels (results not shown), but increased the TEER of monolayers on collagen I gels coated with fibronectin and collagen IV from 215 to 2700 Ω cm^2^ (Fig. 2d, Additional file 1: Figure S2). cAMP had no effect on monolayer formation (data not shown), but also showed a significant increase in TEER, from 215 to 1700 Ω cm^2^ (Fig. 2d), similar to the increase seen with the addition of RI. The combination of cAMP and RI did not result in a further increase in TEER (Fig. 2d).

### Cross-linking

As shown above (Fig. 1a), the coverage of dhBMECs on uncoated collagen I gels increased with increasing stiffness, but did not result in the formation of confluent monolayers even at a concentration 7 mg mL^−1^. While confluent monolayers were formed on 7 mg mL^−1^ gels coated with fibronectin and collagen IV, TEER values were about fivefold lower than on transwell membranes with no gel. To assess whether some of the loss of TEER on gels could be recovered, we used a small molecular cross-linker to increase the gel stiffness. Based on previous work, we estimate that cross-linking results in a fivefold increase in stiffness [29]. Cross-linking did not influence the rate of monolayer formation on 7 mg mL^−1^ gels coated with fibronectin and collagen IV (data not shown), but the TEER increased significantly to 2200 Ω cm^2^. The addition of RI and cAMP did not further increase the TEER.

### Formation of cell–cell junctions

**Fig. 3:**
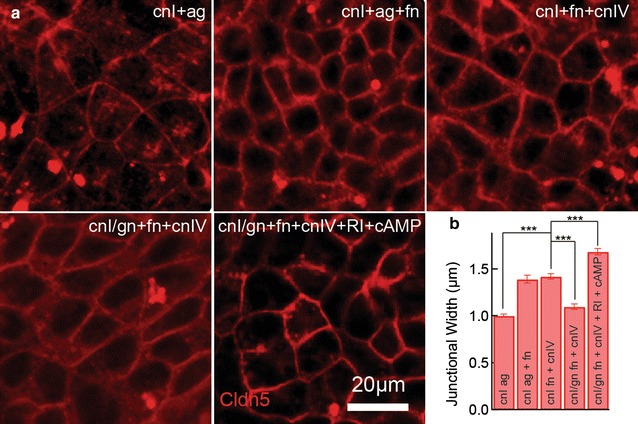
Tight junction formation of dhBMEC monolayers. **a** Maximum intensity projection of claudin-5 confocal z-stack of dhBMECs monolayers on coated collagen I (cnI) and genipin cross-linked collagen I gels. **b** Width of claudin-5 junctions. Bars represent mean ± standard error, *** represents p < 0.001

To confirm the formation of cell–cell junctions on gels that promote monolayer formation, we performed immunofluorescence imaging of zona-occludens 1 (ZO-1) (Additional file 1: Figure S4) and claudin-5 (Fig. 3a). Monolayers formed on gels are similar to monolayers formed on glass but show larger cell areas, consistent with the decrease in adhesion, where fewer cells adhere and hence need to spread out to form junctions with neighboring cells (Additional file 1: Figure S4). Claudin-5-positive junctions were observed on collagen I gels coated with agrin, agrin and fibronectin, and fibronectin and collagen IV, although all conditions show significant intracellular staining. The most distinct claudin-5 networks with the lowest level of intracellular expression were obtained for genipin-treated collagen I gels coated with fibronectin and collagen IV, and with ROCK inhibitor and cAMP added to the media. To gain insight into the degree of cell–cell overlap and the strength of the junction, we used confocal microscopy to determine the width of the claudin-5 junctional stain. Agrin-coated collagen I gels have the narrowest junction width, whereas gels with fibronectin and collagen IV showed significantly wider junctions (Fig. 3b). Although genipin cross-linked gels show low junction width, the incorporation of ROCK inhibitor and cAMP resulted in a significant increase.

### Fabrication of perfusable brain-specific dhBMEC microvessels

**Fig. 4:**
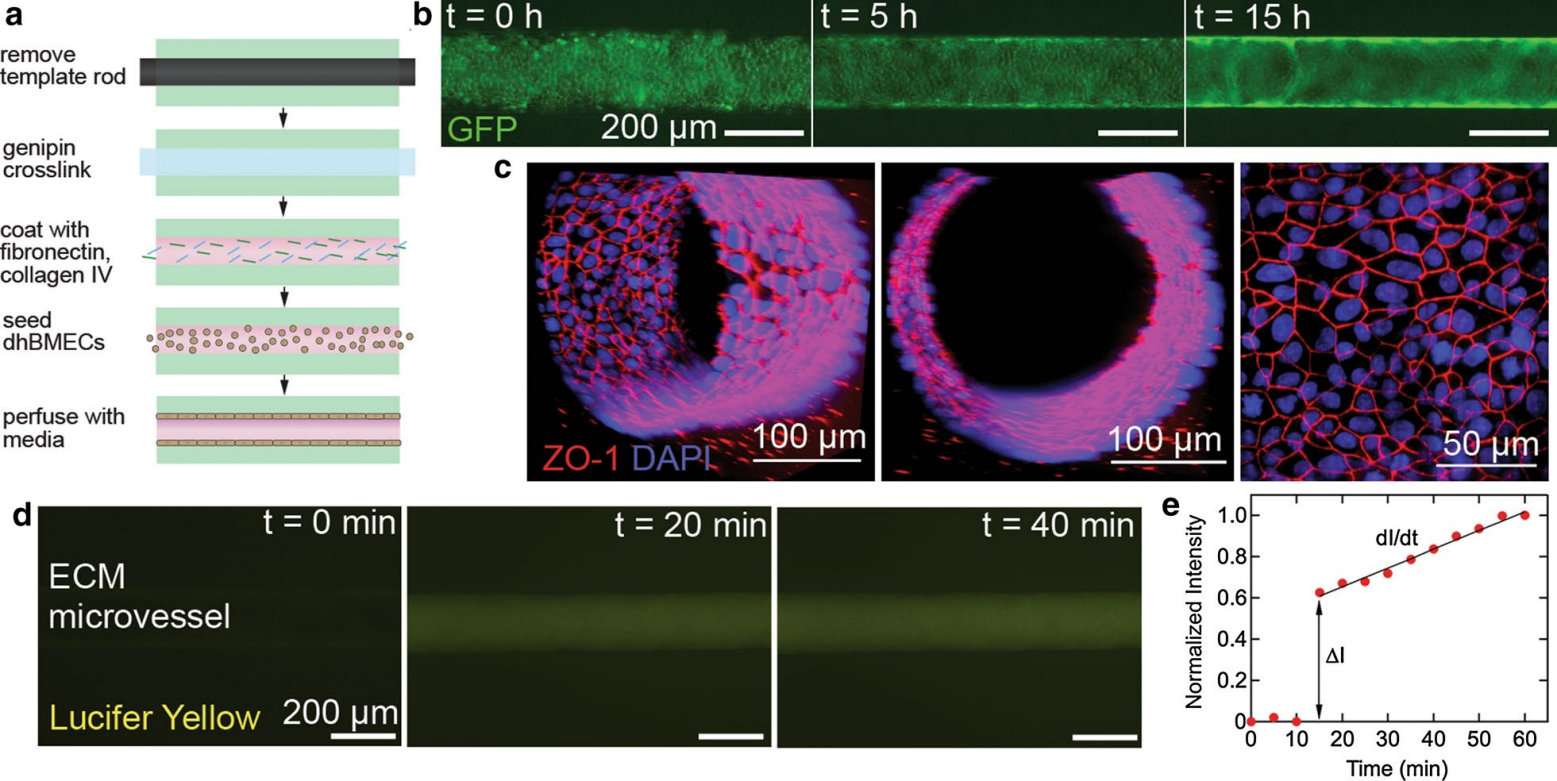
Functional microvessels formed from dhBMECs. **a** Schematic of microvessel construction. **b** BC1 GFP—dhBMEC (GFP) growth in genipin cross-linked 7 mg mL^−1^ collagen gel, coated with collagen IV and fibronectin, with RI, cAMP, Ro-20-1724, and 70 kDa dextran. **c** Confocal image of a dhBMEC microvessel, cross section, and projected image at the bottom of the microvessel. ZO-1 (red), DAPI (blue). **d** Sequence of fluorescence images during a Lucifer yellow permeability experiment. **e** Normalized fluorescence intensity versus time following Lucifer yellow introduction into the microvessel

The ability to derive hBMECs with BBB phenotype from iPSCs provides the opportunity to develop a new generation of tissue-engineered BBB models. dhBMECs form monolayers with expression of tight junction markers and very high TEER on glass and transwell membranes [8]. However, compared to other ECs commonly used in tissue engineering, formation of dhBMEC monolayers is much more difficult on ECM materials, representing a major barrier to the integration of these cells in 3D BBB models. Having screened gel concentrations, coatings, and soluble factors, we next used these results to form dhBMEC microvessels (Fig. 4a).

We first seeded GFP-labeled dhBMECs in cylindrical channels within a 7 mg mL^−1^ collagen I matrix coated with collagen IV and fibronectin. Cells seeded under these conditions resulted in poor adhesion and death of the majority of cells over the first 24 h, as evidenced by blebbing and loss of intracellular fluorescence (data not shown). Using fibronectin and/or agrin to coat the vessels did not significantly increase coverage or cell viability. To overcome the low density of adherent cells, sequential seeding, with cells seeded every 3 days, resulted in higher cell densities in the microvessel, although not sufficient for microvessel formation.

The addition of ROCK inhibitor to the media reduced the large amount of cell death in the first 24 h of culture following seeding in channels. In conjunction with sequential seeding, the addition of ROCK inhibitor resulted in nearly confluent microvessels. The inclusion of cAMP had a similar result, but by different means. Rather than inhibiting cell death, cAMP increased the rate of adhesion and spreading, as reported elsewhere [37]. Combining ROCK inhibitor and cAMP into the media made moderate improvements, with the number of cells adhering to the collagen increasing, but still unable to form confluent microvessels (data not shown).

Cross-linking the collagen I gel proved essential for microvessel formation. Following cross-linking with genipin, fully confluent microvessels were formed within 24 h (Fig. 4b). Functional dhBMEC microvessels were formed in genipin cross-linked gels coated with collagen IV and fibronectin, and with RI and cAMP in the culture media. Phosphodiesterase inhibitor Ro-20-1724 and 70 kDa dextran were also incorporated into the culture medium to promote long term vessel stability, as adhesion and survival were no longer the primary concern [30]. Using these conditions functional microvessels were maintained for more than 1 week with a single seeding.

Confocal images of the dhBMEC microvessels show a network of well-defined cell–cell junctions (Fig. 4c). The cells do not elongate and align under flow, as we have shown previously [38].

To assess barrier function we measured the permeability of Lucifer yellow, a small anionic salt (Fig. 4d) [39]. The flow rate was about 200 µL h^−1^ corresponding to a shear stress of about 1 dyn cm^−2^, typical of post-capillary venules [1]. The permeability was calculated from the fluorescence intensity using standard methods developed for microvessels (Fig. 4e) [32]. The Lucifer yellow permeability in the dhBMEC microvessels was 0.70 ± 0.21 × 10^−6^ cm s^−1^, similar to the value of 0.37 ± 0.11 × 10^−6^ cm s^−1^ obtained for dhBMEC monolayers using the transwell assay [8]. This permeability value is much lower than that reported in other brain microvessel models with permeability values over an order of magnitude higher using larger molecular probes [27] and in HUVEC microvessels [22]. In addition to showing very low permeability, the microvessels also showed no detectable focal leaks. Focal leaks are characterized by localized plumes of increased fluorescence, and were not observed with the 5 min imaging frequency. Focal leaks of much larger fluorescently-labeled probes have been observed in microvessels formed from HUVECs and HMVECs at this imaging frequency [22, 33]. The fact that we observe no characteristic focal leaks with a small molecule further supports the formation of physiological tight junctions.

## Discussion

### Cell adhesion

The interstitial space in the brain includes a complex ECM composed of hyaluronic acid, laminin, proteoglycans, and tenascins [40–43]. In vitro models of the BBB need to take into account the brain ECM, but also must meet additional requirements; an ECM material must provide structural support and chemical cues to maintain barrier function. We considered hyaluronic acid (HA), fibrin, and collagen I, three structural materials commonly used in tissue engineering. In preliminary work we showed that dhBMECs exhibit very poor adhesion on HA and hence this matrix material was not considered further. Fibrin, another common structural hydrogel used in microvessel models [44], is rapidly digested by BMECs and hence was not a candidate matrix material. Therefore, we selected collagen I as the structural matrix for our BBB model. Collagen I is the most prevalent structural protein in the body, and is widely used in in vitro models [22, 25, 45], but is not present in the brain [43]. However, collagen I provides a stable, structural matrix that can be modified to promote adhesion, spreading of dhBMECs and promote barrier function. In previous work we have also shown that gels based on collagen I can recapitulate the morphology and low levels of GFAP expression associated with quiescent astrocytes in the human brain [40], providing a structural support that mimics the stiffness of the brain.

Matrix stiffness is known to play an important role in promoting adhesion and spreading of endothelial cells and other cell types [46–50]. Here we show that dhBMECs form mounds on softer collagen I gels, but spread and form confluent regions on 6 and 7 mg mL^−1^ collagen I gels. The collagen I concentrations correspond to a stiffness of 189 ± 4, 1840 ± 76, and 3800 ± 162 Pa for 4, 6, and 8 mg mL^−1^ respectively, based on AFM measurements reported previously [40], and in agreement with previous reports in the literature [51]. The bulk shear modulus for the human, rat, and pig brain is around 200–300 Pa [35, 52], corresponding to around 4 mg mL^−1^ collagen. The monotonic increase in dhBMEC coverage with stiffness suggests that other factors are important in establishing the endothelium in vivo, compared to the relative importance of bulk stiffness in vitro.

Brain capillaries and microvessels are surrounded by a basement membrane, which is composed of laminin, fibronectin, collagen IV, and heparan sulfate proteoglycans agrin, perlecan, and collagen XVIII [53–55]. The makeup of the BMEC basement membrane is unique and critical for BMEC barrier formation [56–60]. Here we investigated the role of fibronectin, laminin, collagen IV, perlecan, and agrin as surface coatings to mimic components of the basement membrane in the brain. The addition of basement membrane proteins fibronectin, collagen IV, and laminin promoted adhesion and spreading. This result is consistent with similar experiments using primary porcine cerebral endothelial cells [53]. The addition of agrin and perlecan, two heparin sulfate proteoglycans present in the basement membrane in the BBB, resulted in very different responses. While perlecan had no apparent influence on adhesion and spreading, agrin had the largest effect in promoting adhesion and spreading. Agrin loss has been associated with BBB dysfunction and breakdown, and agrin coating has been reported to decrease permeability, proliferation, and increase junctional staining of murine bEnd5 cells [61]. Although agrin promoted adhesion, monolayers showed weak claudin-5 junctional staining and agrin abolished barrier function.

Cell–cell overlap may be important for BMECs as it likely enhances the formation of stable tight junctions that keep the brain isolated from circulation. Evidence of large cell–cell overlap has been inferred from 2D shear stress experiments, where the cells do not migrate far from their starting position when compared to HUVECs [17]. We hypothesize that larger cell–cell overlap helps to increase and maintain barrier function. In our indirect measurement of cell–cell overlap we found that cells plated on 7 mg mL^−1^ collagen I gels crosslinked with genipin, coated with collagen IV and fibronectin, and cultured with ROCK inhibitor and cAMP, show evidence of significantly more cell–cell overlap. Laminin/entactin was selected as an additional ECM component as it is present in the developing brain and has been shown to be an important additive to ECM to support the maintenance of endothelial cells [40, 54]. No change in adhesion was observed with the addition of laminin/entactin into the ECM.

### Barrier function

TEER values for dhBMEC monolayers decreased from 5500 Ω cm^2^ on transwell membranes coated with fibronectin and collagen IV, to 215 Ω cm^2^ on 7 mg mL^−1^ collagen gels coated with fibronectin and collagen IV, highlighting the important role of substrate stiffness on adhesion and barrier function. This high TEER value is consistent with recent publications using derived hBMECs in monoculture [14, 62], and is approaching the calculated electrical resistance of brain microvessels of around 8000 Ω cm^2^ [63], and significantly higher than other hBMEC cell lines, which are typically ≤ 200 Ω cm^−2^ [64–66]. The addition of agrin promoted monolayer formation (Fig. 1d), but down-regulated barrier function (Fig. 2c), illustrating that improved adhesion and spreading does not necessarily predict improved barrier function. However, the addition of fibronectin restored barrier function. The origin of this effect is beyond the scope of this study, however, we speculate that agrin promotes the formation of focal adhesions to the detriment of cell–cell junctions.

Treatment with ROCK inhibitor, cAMP, or cross-linking with genipin resulted in recovery of the TEER on bulk gels to about 2500 Ω cm^2^, about half of the value on transwell membranes (no gel). Combinations of treatment with ROCK inhibitor, cAMP, or cross-linking also resulted in TEER values of around 2500 Ω cm^2^ for all dhBMEC monolayers. ROCK inhibitor is commonly used to prevent apoptosis in embryonic and induced pluripotent stem cell cultures [67] and has been shown to play a role in the adhesion of cancer cells [68]. In addition, ROCK inhibitor has been shown to enhance survival, adhesion, and TEER following cryopreservation of dhBMECs, possibly by decreasing cellular stress as measured by actin stress fiber formation [69]. cAMP is well known to contribute to barrier function in the BBB [70, 71], and improve barrier function in monolayers and in vitro microvessel models [23, 37].

We speculate that the maximum TEER values for monolayers on gels (around 2500 Ω cm^2^) are lower than for dhBMECs on transwell inserts (around 5500 Ω cm^2^) due to the relatively rough surface morphology of the gels and the resulting difficulty in forming a good seal with the sidewalls at the perimeter. Claudin-5 positive tight junctional networks were formed using a number of the gel conditions, including genipin cross-linked collagen I gels with ROCK inhibitor and cAMP. In summary, these results show that monolayer formation, barrier function, and tight junction formation can be achieved in cross-linked collagen I gels coated with fibronectin and collagen IV, and treated with ROCK inhibitor and cAMP. These results provide key insight into strategies for tissue engineering perfusable dhBMEC microvessels. ROCK inhibitor does not increase the number of cells that adhere to the matrix, but rather the number of cells that survive the first 24 h and thus remain adhered. On collagen I gels, cell adhesion and spreading typically takes 24–48 h [72]. ROCK inhibitor may allow the cells more time to form these junctions before initiating apoptosis, just as it does in iPSC cultures, and allow the cells more time to form cadherin junctions with neighboring cells [67].

### dhBMEC microvessels

Having identified matrix compositions and coatings that promote adhesion, spreading, and barrier function, we have successfully applied these conditions to the formation of functional brain-specific microvessels. Tissue-engineered models of the BBB have been reported using primary rat [26], immortalized human [28], and primary human [27] brain microvascular endothelial cells. All of these models show apparent permeabilities on the order of 10^−6^ cm s^−1^ for large molecular weight (3–40 kDa) dextran. In one study, the addition of astrocytes in the surrounding matrix resulted in a permeability of 0.6 × 10^−6^ cm s^−1^ for 4 kDa dextran [28]. Permeability to high molecular weight dextrans is typical for tumor vasculature, but is not physiological in the BBB. Here we show an apparent permeability of 0.7 × 10^−6^ cm s^−1^ for Lucifer yellow (MW 442), similar to values obtained in the transwell assay. Furthermore, in contrast to microvessels using other endothelial cell types, we detect no transient focal leaks. These results suggest that microvessels formed from dhBMECs can mimic the barrier function of the BBB.

## Conclusions

To assess the parameters that control adhesion, spreading, and survival of dhBMECs, we systematically screened matrix stiffness (collagen I, crosslinking with genipin), compositions (laminin/entactin), protein coatings (fibronectin, laminin, collagen IV, perlecan, and agrin), and soluble factors in the culture medium (ROCK inhibitor and cyclic adenosine monophosphate). Increasing matrix stiffness increased adhesion but did not result in monolayer formation. Coating 7 mg mL^−1^ collagen I gels with fibronectin, collagen IV, and laminin resulted in high coverage but unreliable monolayer formation. However, coating collagen I gels with agrin, with or without fibronectin and collagen IV, resulted in monolayer formation. The addition of ROCK inhibitor or cAMP to the culture medium, or gel cross-linking, resulted in a significant increase in TEER to around 2500 Ω cm^2^. Based on these results, we successfully fabricated dhBMEC microvessels in cross-linked collagen I gels coated with fibronectin and collagen IV, and treated with ROCK inhibitor and cAMP. We measured apparent permeability values for Lucifer yellow, comparable to values obtained in the transwell assay. During these experiments we observed no focal leaks, suggesting the formation of tight junctions that effectively block paracellular transport.

## Additional file

**Additional file 1.** Supplementary information, containing description of junctional width measurement, 2D adhesion assay, representative TEER values, and cell area on collgen I and glass substrates.

## Abbreviations

AFM: atomic force microscopy
ag: agrin
BBB: blood–brain barrier
BMEC: brain microvascular endothelial cell
cAMP: cyclic adenosine monophosphate
cnI: collagen I
cnI/gn: genipin treated collagen I
cnIV: collagen IV
dhBMEC: derived human brain microvascular endothelial cell
EC: endothelial cell
ECM: extracellular matrix
fn: fibronectin
HA: hyaluronic acid
hBMEC: human brain microvascular endothelial cell
hiPSC: human induced pluripotent stem cell
HMVEC: human microvascular endothelial cells
HUVEC: human umbilical vein endothelial cells
iPSC: induced pluripotent stem cell
KOSR: knockout serum replacement
ln: laminin
PBS: phosphate buffered saline
pc: perlecan
RI: rho-associate kinase inhibitor
ROCK: rho-associated kinase
TEER: transendothelial electrical resistance
ZO-1: zona occludin 1
