# Supplementary material for: Functional brain-specific microvessels from iPSC-derived human brain microvascular endothelial cells: the role of matrix composition on monolayer formation
Source: Fluids Barriers CNS. 2018 Feb 20;15:7. doi: 10.1186/s12987-018-0092-7 (PMC5819713; doi:10.1186/s12987-018-0092-7)
Supplement: Supplementary file 1 — Additional file 1. Supplementary information, containing description of junctional width measurement, 2D adhesion assay, representative TEER values, and cell area on collgen I and glass substrates. [file 12987_2018_92_MOESM1_ESM.docx]

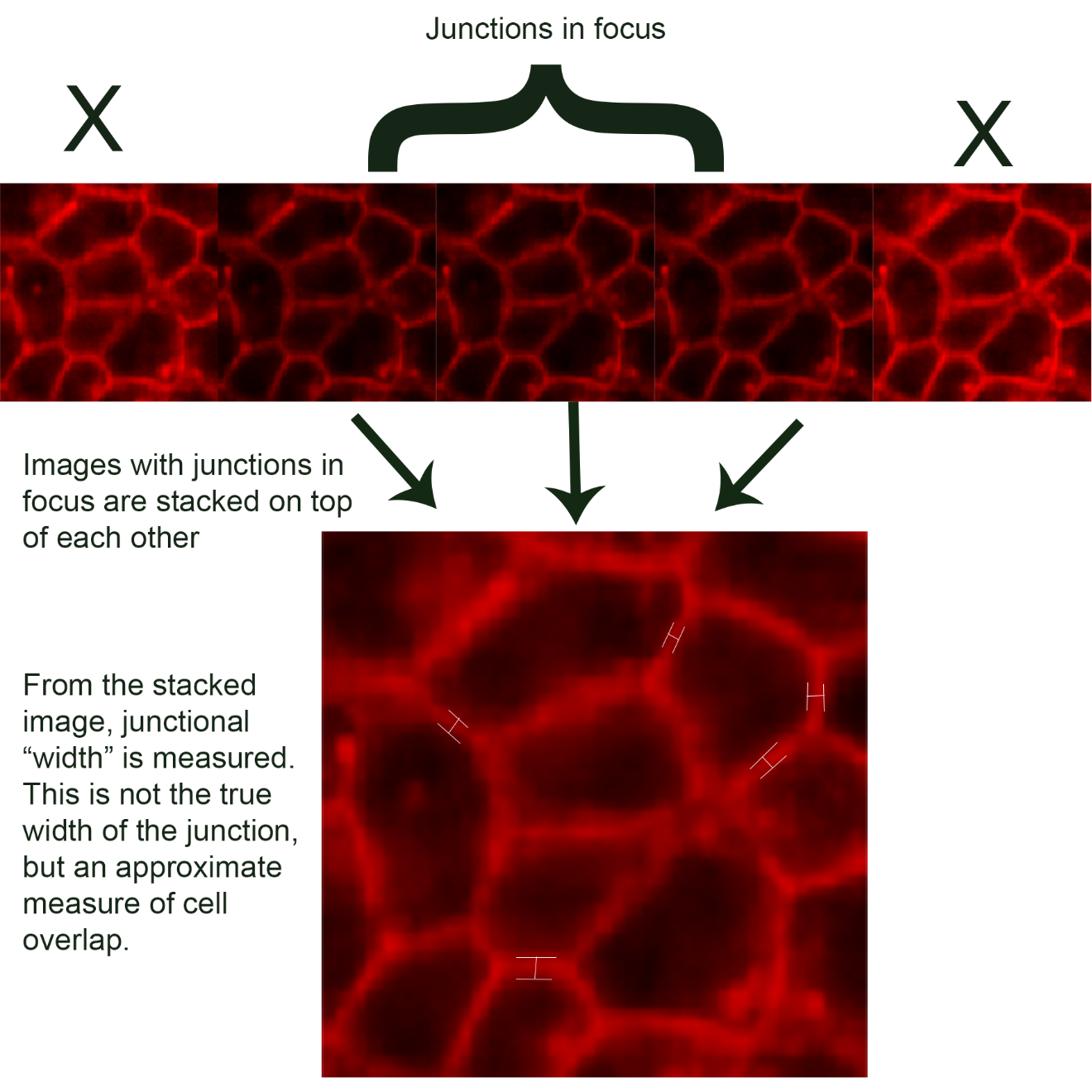


**Figure S1: Junctional width measurement.** Schematic representation of junctional width measurement. Images from the z-stack are observed to determine if the junctions are in focus, all images with sharp in focus junctions are then stacked. The width of the junctional stain was measured as an indicator of cell overlap.

**
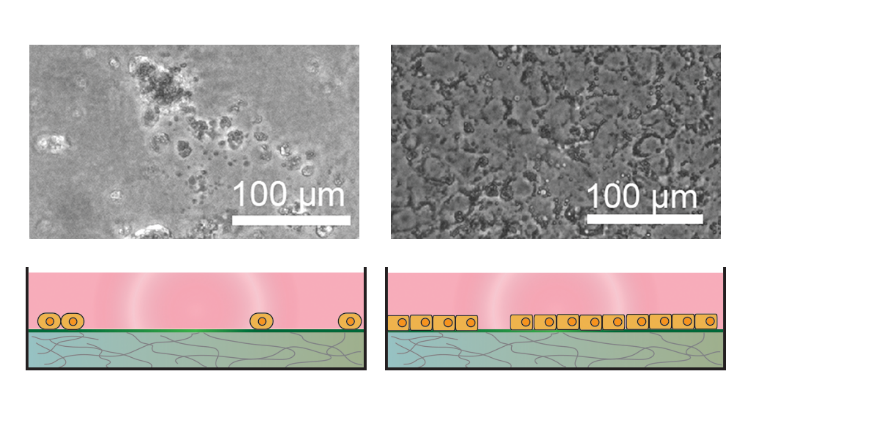
**

**Figure S2:** Representative images of 2D adhesion assay showing the two main outcomes.

**Figure S3:**  Representative TEER values for one gel condition over the course of one week.


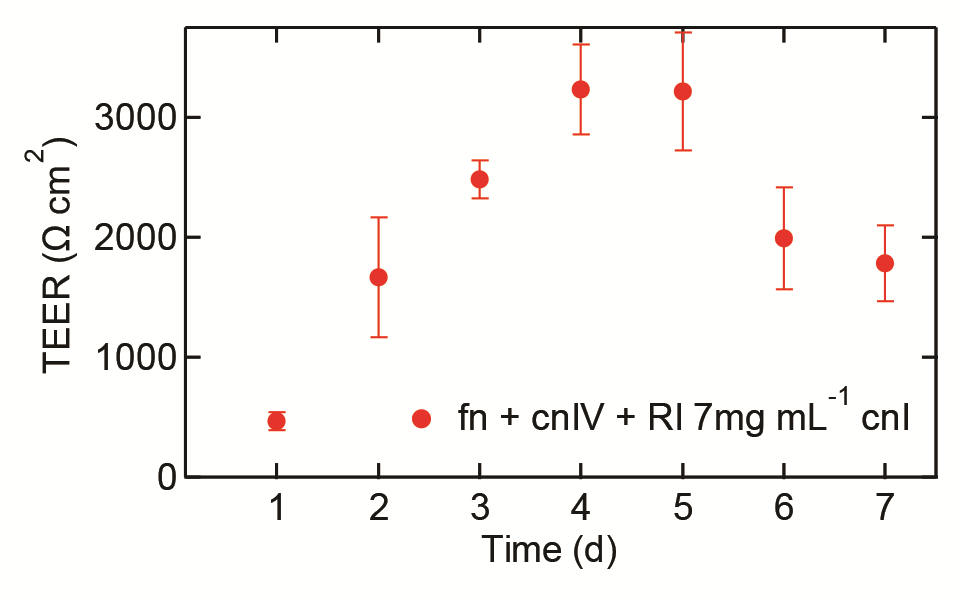

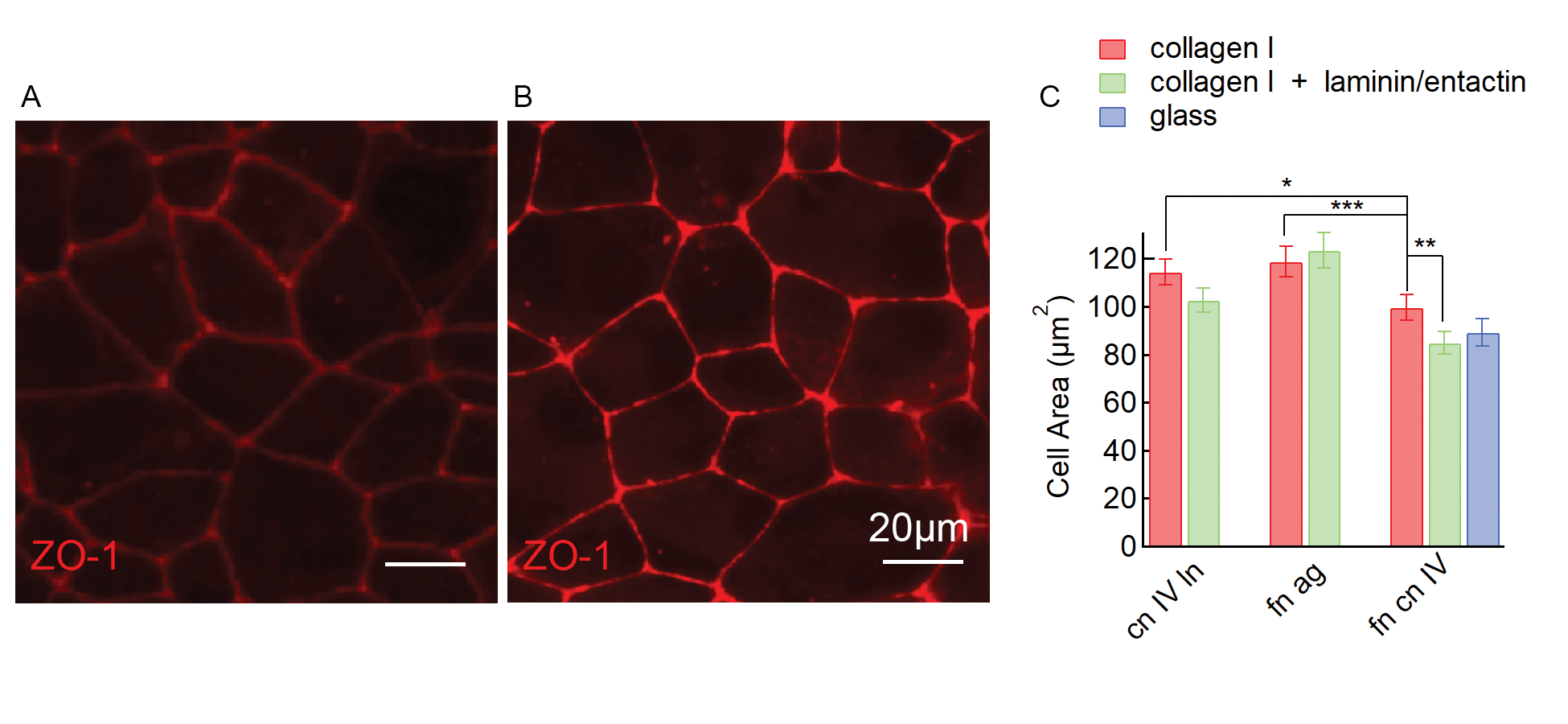


**Figure S4:** **Junctional formation and analysis on coated gels.** (A) ZO-1 stain on 7 mg mL^-1^ collagen I gel coated with fibronectin and collagen IV. (B) ZO-1 stain on fibronectin and collagen IV coated glass. (C) cell area analysis for gel and glass coatings.
